# Microgravity Spherical Droplet Evaporation and Entropy Effects

**DOI:** 10.3390/e25081232

**Published:** 2023-08-18

**Authors:** Seyedamirhossein Madani, Christopher Depcik

**Affiliations:** Department of Mechanical Engineering, University of Kansas, Lawrence, KS 66045, USA; depcik@ku.edu

**Keywords:** droplet evaporation, microgravity, low-temperature combustion, entropy generation

## Abstract

Recent efforts to understand low-temperature combustion (LTC) in internal combustion engines highlight the need to improve chemical kinetic mechanisms involved in the negative temperature coefficient (aka cool flame) regime. Interestingly, microgravity droplet combustion experiments demonstrate this cool flame behavior, allowing a greater focus on chemistry after buoyancy, and the corresponding influence of the conservation of momentum is removed. In Experimental terms, the LTC regime is often characterized by a reduction in heat transfer losses. Novel findings in this area demonstrate that lower entropy generation, in conjunction with diminished heat transfer losses, could more definitively define the LTC regime. As a result, the simulation of the entropy equation for spherical droplet combustion under microgravity could help us to investigate fundamental LTC chemical kinetic pathways. To provide a starting point for researchers who are new to this field, this effort first provides a comprehensive and detailed derivation of the conservation of entropy equation using spherical coordinates and gathers all relevant information under one cohesive framework, which is a resource not readily available in the literature. Subsequently, the well-known d^2^ law analytical model is determined and compared to experimental data that highlight shortcomings of the law. The potential improvements in the d^2^ law are then discussed, and a numerical model is presented that includes entropy. The resulting codes are available in an online repository to ensure that other researchers interested in expanding this field of work have a fundamental starting point.

## 1. Introduction

Practical combustion systems often rely on liquid fuel droplet evaporation and ignition for power delivery and heat release. Thus, the analysis of these droplets can impact system performance, emission reduction, and process characterization. Typically, harmful emissions that emanate from liquid fuel combustion, such as particulate matter (PM) and nitrogen oxides (NOx), are limiting factors during conventional combustion modes in internal combustion engines [1,2]. PM is generated as a result of the presence of regions with an excess of fuel, while NOx is produced under lean conditions (i.e., an excess of air) when the temperature is sufficient to break the triple bond of atmospheric nitrogen. Whereas PM can be reduced via combustion at higher temperatures, a reduction in NOx requires lower temperatures, indicating a trade-off between these two problematic emissions. In-cylinder strategies, such as the use of exhaust gas recirculation, have been implemented to decrease these emissions, as has the utilization of after-treatment devices. However, the widespread use of after-treatment systems is hindered by increased economic costs, challenges related to durability, decreased fuel efficiency, and the space required on-board the vehicle to house the complete after-treatment system [3]. The result of which has directed researchers into investigating low-temperature combustion (LTC), which is a combustion regime that occurs at relatively lower temperatures than traditional combustion modes. In LTC, the air–fuel mixture is intentionally ultra-lean and lowers peak temperatures below the nitrogen bond breaking point, hence reducing the production of NOx. In parallel, LTC aims to employ a homogenous air–fuel mixture to decrease the number of fuel-rich pockets, which mitigates PM formation [4,5]. LTC is achieved through various techniques, such as homogeneous charge compression ignition (HCCI), pre-mixed charged compression ignition (PCCI), and reactivity-controlled compression ignition (RCCI). The aim of realizing LTC operation is to obtain higher fuel efficiencies and lower emissions by optimizing the combustion process under low temperatures and lean conditions [6].

With respect to liquid fuel combustion during LTC mode operation, the ignition of alkanes, which are primary components of ultra-low sulfur diesel, often involves understanding the “cool flame” phenomenon, along with each fuel’s negative temperature coefficient (NTC) behavior. The formation of a cool flame and its spread involve identifying the influence of heat transfer [7], whereas the NTC region is indicative of the increasing temperature range over which the reaction kinetics slow down, which subsequently leads to the development of a quasi-steady flameless combustion process [8]. Beyond the NTC region, sustained high-temperature combustion can be realized at increasing temperatures. This two-stage ignition process (cool and then hot flames) has been observed in microgravity experiments carried out on the International Space Station or at ground-bases facilities [9,10,11]. For relatively large droplets, the hot ignition stage is extinguished through radiative heat losses, which then form a cool flame that lies closer to the droplet. This cool flame can be sustained through the heat release from NTC reaction kinetics and the loss of heat through diffusion.

The use of a microgravity environment can simplify the study of droplet evaporation and combustion by providing a unique spherical symmetry configuration. In normal gravity conditions, buoyancy affects the vaporization and burning of the droplet by causing an upward advective flow in the proximity of the droplet. In contrast, in microgravity conditions, the phenomena, including droplet heat-up, gas and liquid transport, evaporation, multiphase physics, and reaction kinetics, can be independently investigated [12,13]. As a result, the influence of the conservation of momentum equation is removed, and it does not need to be solved as part of the solution methodology. The first models related to droplet evaporation and combustion were developed in the 1950s and culminated in the introduction of the d^2^ law [14,15]. This “law” is an analytical model that relates the droplet vaporization rate to the square of the droplet diameter. This model has been found to offer reasonably precise predictions for a wide range of droplet sizes and conditions. However, it is far from complete, as it only considers the conservation equations for the constant-property gaseous phase. Subsequently, the first experiments on microgravity were carried out a few years later [16], and detailed models of isolated droplet evaporation and combustion have been presented ever since, which emphasize different aspects, such as droplet heat-up, flame stability, quasi-steady burning, extinction, radiation, and spray configuration [17,18,19,20]. During the 1990s, microgravity experiments were able to distinctly identify the LTC operating mode, thus expanding their pertinence [21,22]. Moreover, several numerical models are in good agreement with microgravity combustion experimental data, highlighting the necessity of incorporating LTC kinetics to accurately predict ignition delays, explosion patterns, and burning behaviors [23,24,25].

While LTC has garnered a great deal of attention as a potential future means of emissions control, these strategies are characterized by greater fuel consumption and reduced thermal efficiency, especially at high engine loads. Thus, expanding the operating range of LTC while maintaining its benefits is an ongoing research area [26]. It has recently been indicated in the literature that the second law of thermodynamics can act as a useful tool to identify LTC regions and can provide a potential means of improving overall efficiencies by analyzing the availabilities and irreversibilities of the system. Indeed, lower entropy generation in conjunction with diminished heat transfer losses could more definitively define the LTC regime [27,28,29,30]. In this regard, Mahabadipour et al. [31,32] used a dual fuel diesel–natural gas LTC engine model to study entropy and exergy to quantify thermodynamic irreversibilities, as well as entropy generation. They were able to measure the destroyed exergy in different zones within the engine and identified the contributions of different phenomena, such as combustion, heat transfer, and work. Xu et al. [33] employed computational fluid dynamics simulations to investigate the combustion characteristics of a heavy-duty compression ignition engine that adopted LTC. The study emphasized the implementation of energy and exergy analyses by examining different engine parameters and their impacts on thermal efficiency, emissions, and exergy destruction. Liu et al. [34] focused on the analysis of exergy destruction mechanisms in LTC-related conditions. They also proposed a qualitative exergy loss map based on equivalence ratio and temperature, highlighting that the LTC of products generated through rich fuel/air reforming can minimize exergy destruction and mitigate NOx and PM formation. Shirvani et al. [35] investigated different LTC schemes using both the first and second laws of thermodynamics, evaluating them against the ideal constant pressure cycle and a conventional combustion process. They determined that HCCI and RCCI exhibit the greatest exergy efficiency and the lowest exergy destruction. 

While a few studies have incorporated the concept of entropy generation to understand entropy changes and investigate the efficiency of the internal combustion engines, a comprehensive and detailed derivation of the entropy balance equation for reacting flows remains scarce in the existing literature. Additionally, the specific contributions of each process to the entropy equation, as well as their inter-relationships, remain unclear when examining single-droplet evaporation and combustion in microgravity. Thus, this effort provides the derivation of the conservation of entropy in spherical coordinates for reacting flows. Subsequently, the d^2^ law analytical model is provided and compared to existing experimental data in the literature, and we highlight its shortcomings. Next, a numerical model for the analysis of the fuel droplet evaporation process, including the entropy equation, is introduced by utilizing the d^2^ law assumptions. Overall, this effort provides an appropriate starting point for a researcher new to the field of microgravity combustion and the conservation of entropy to expand their knowledge.

## 2. Entropy Balance Equation Derivation for Reacting Flows

**Figure 1:**
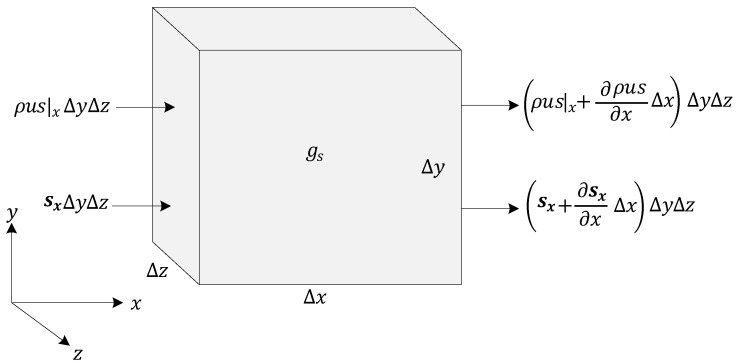
Terms in the entropy–flux balance for a 3D fluid element.

In a multicomponent gas mixture with N number of species, we considered an arbitrary differential fluid element with a volume of Δx·Δy·Δz and constant dimension sizes, as illustrated in Figure 1. For this control volume (CV), several factors contribute to the balance of the entropy:

Firstly, we considered the rate of accumulation of entropy in the CV as:

(1) $$\frac{\partial \left(\rho s\right)}{\partial t}\;\Delta x\;\Delta y\;\Delta z$$

where ρ is the density of the mixture [kg m^−3^], s is the entropy per unit mass of the mixture [m^2^ s^−2^ K^−1^], and t is time [s]. The rate of influx of entropy to the CV due to advection in the x-direction at x is defined as follows:

(2) $$\rho us{|}_{x}\Delta y\Delta z$$

where u is the x-directional component of velocity [m s^−1^]. The rate of outflux of entropy from the CV due to advection in the *x*-direction at x+Δx is defined as follows:

(3) $$\rho us{|}_{x+\Delta x}\Delta y\Delta z=\rho us{|}_{x}\Delta y\Delta z+\frac{\partial \left(\rho us\right)}{\partial x}\Delta x\Delta y\Delta z$$

Thus, by incorporating a negative sign via Equation (3) to account for the idea that entropy into the CV is positive, the net rate of entropy flux within the CV due to advection in the x-direction is defined as follows:

(4) $$-\frac{\partial \left(\rho us\right)}{\partial x}\Delta x\Delta y\Delta z$$

While the entropy change within the CV is dependent upon advection and accumulation, the entropy balance equation includes an entropy flux vector s [J m^−2^ K^−1^ s^−1^], which represents the reversible entropy flow, as well as the irreversible generation of entropy per unit volume $g_{s}$ [J m^−3^ K^−1^ s^−1^]. By considering the inputs and outputs of the entropy in y and z directions and using the method given by Hirschfelder et al. [36], the entire entropy balance within the CV when divided by the element volume will be as follows:

(5) $$\frac{\partial \rho s}{\partial t}=-\left(\frac{\partial \left(\rho us\right)}{\partial x}+\frac{\partial \left(\rho vs\right)}{\partial y}+\frac{\partial \left(\rho ws\right)}{\partial z}\right)-\nabla \cdot s+g_{s}$$

where v and w are y- and z-directional components of the velocity, respectively. Expanding the first and second terms in the left-hand side of Equation (5) gives the following equation:

(6) $$\frac{\partial \rho s}{\partial t}+\nabla \cdot \left(\rho sV\right)=\rho \frac{\partial s}{\partial t}+s\frac{\partial \rho }{\partial t}+s\nabla \cdot \left(\rho V\right)+\rho V\cdot \nabla s$$

where V, which is the velocity vector, is defined as $V=u\;\hat{x}+v\;\hat{y}+w\;\hat{z}$. The sum of second and third terms on the right-hand side of the Equation (6) is the continuity equation, which equals zero. Thus, Equation (6) can be represented as follows:

(7) $$\rho \left(\frac{\partial s}{\partial t}+u\frac{\partial s}{\partial x}+v\frac{\partial s}{\partial y}+w\frac{\partial s}{\partial z}\right)=-\nabla \cdot s+g_{s}$$

In Equation (7), entropy is a function of the density, chemical composition, and temperature of the gaseous phases and can be directly calculated through known variables in the conservation equations for mass, species, and energy, which are presented as follows [37]:

(8) $$\frac{D\rho }{Dt}+\rho \nabla \cdot V=0$$

(9) $$\rho \frac{DY_{i}}{Dt}={\dot{\omega }}_{i}-\nabla \cdot \left(\rho Y_{i}\vartheta_{i}\right)$$

(10) $$\rho \frac{De}{Dt}=-p\left(\nabla \cdot V\right)-\nabla \cdot q+\Phi +\rho \sum_{i=1}^{N}Y_{i}f_{i}\vartheta_{i}$$

where $Y_{i}$ and $\vartheta_{i}$ are the mass fraction [-] and diffusion velocity [m s^−1^] of species i, respectively, $f_{i}$ is the external force per unit mass [m s^−2^] exerted on species i, and ${\dot{\omega }}_{i}$ [kg m^−3^ s^−1^] is the rate of consumption or production of species i due to chemical reactions. In Equation (10), e is the specific internal energy [J kg^−1^], p is the mixture pressure [Pa], Φ is the contribution of the viscosity and strain rate tensor to the energy conservation equation, and q is the heat flux vector per unit surface area [W m^−2^]. In this expression, D indicates the definition of substantial derivative, which is a derivative taken along a path moving with velocity V and presented as follows:

(11) $$\frac{D()}{Dt}=\frac{\partial ()}{\partial t}+u\frac{\partial ()}{\partial x}+v\frac{\partial ()}{\partial y}+w\frac{\partial ()}{\partial z}$$

The relationship between the change in specific internal energy and the change in specific entropy can be expressed using Gibbs’ equation as follows:

(12) $$Tds=de+pd\left(\frac{1}{\rho }\right)-\sum_{i=1}^{N}\mu_{c,i}dY_{i}$$

where T is the mixture temperature [K], and $\mu_{c}$ is the specific chemical potential [J kg^−1^]. Equation (12) is obtained by combining the first and second laws of thermodynamics for systems with matter exchange (open systems), in which internal energy is not only a function of entropy and volume, but also a function of the amount of substances. For the detailed derivation of open systems, we referred to the work of Knuiman et al. [38]. Equation (12) was applied to any differential change; thus, after employing a substantial derivative, we found the following equation:

(13) $$T\frac{Ds}{Dt}=\frac{De}{Dt}-\frac{p}{\rho^{2}}\frac{D\rho }{Dt}-\sum_{i=1}^{N}\mu_{c,i}\frac{DY_{i}}{Dt}$$

Substituting Equations (8)–(10) into Equation (13) gives the following equation:

(14) $$\rho \frac{Ds}{Dt}=-\frac{1}{T}\nabla \cdot q+\frac{\Phi }{T}+\frac{\rho }{T}\sum_{i=1}^{N}Y_{i}f_{i}\vartheta_{i}-\frac{1}{T}\sum_{i=1}^{N}\mu_{c,i}\left({\dot{\omega }}_{i}-\nabla \cdot \left(\rho Y_{i}\vartheta_{i}\right)\right)$$

Expanding the identities yields the following equations:

(15) $$-\frac{1}{T}\nabla \cdot q=-\nabla \cdot \left(\frac{q}{T}\right)-\frac{q}{T^{2}}\nabla T$$

and

(16) $$\frac{1}{T}\sum_{i=1}^{N}\mu_{c,i}\nabla \cdot \left(\rho Y_{i}\vartheta_{i}\right)=\nabla \cdot \frac{1}{T}\sum_{i=1}^{N}\left(\mu_{c,i}\rho Y_{i}\vartheta_{i}\right)+\frac{1}{T^{2}}\sum_{i=1}^{N}\left(\mu_{c,i}\rho Y_{i}\vartheta_{i}\right)\nabla T-\frac{1}{T}\sum_{i=1}^{N}\rho Y_{i}\vartheta_{i}\nabla \left(\mu_{c,i}\right)$$

Substituting Equations (15) and (16) into Equation (14) gives the following equation:

(17) $$\rho \frac{Ds}{Dt}=-\nabla \cdot \left[\left(\frac{q}{T}\right)-\frac{1}{T}\sum_{i=1}^{N}\left(\mu_{c,i}\rho Y_{i}\vartheta_{i}\right)\right]-\frac{q}{T^{2}}\nabla T+\frac{\Phi }{T}+\frac{\rho }{T}\sum_{i=1}^{N}Y_{i}f_{i}\vartheta_{i}-\frac{1}{T}\sum_{i=1}^{N}\mu_{c,i}{\dot{\omega }}_{i}+\frac{1}{T^{2}}\sum_{i=1}^{N}\left(\mu_{c,i}\rho Y_{i}\vartheta_{i}\right)\nabla T-\frac{1}{T}\sum_{i=1}^{N}\rho Y_{i}\vartheta_{i}\nabla \left(\mu_{c,i}\right)$$

Comparing Equations (7) and (17) leads to the following expressions:

(18) $$s=\left(\frac{q}{T}\right)-\frac{1}{T}\sum_{i=1}^{N}\left(\mu_{c,i}\rho Y_{i}\vartheta_{i}\right)$$

and

(19) $$g_{s}=-\frac{q}{T^{2}}\nabla T+\frac{\Phi }{T}+\frac{1}{T}\sum_{i=1}^{N}\rho Y_{i}\vartheta_{i}\left(f_{i}+\frac{\mu_{c,i}}{T}\nabla T-\nabla \mu_{c,i}\right)-\frac{1}{T}\sum_{i=1}^{N}\mu_{c,i}{\dot{\omega }}_{i}$$

Equation (19) indicates the respective contributions of heat transfer, viscous dissipation, mass transfer, and chemical reactions to the generation of entropy. The total heat flux can be obtained by combining the individual heat flux parameters as follows:

(20) $$q=-\lambda \nabla T+\sigma_{b}\varepsilon T^{4}+R_{u}T\sum_{i=1}^{N}\sum_{j=1}^{N}\frac{X_{j}D^{T}}{m_{i}D_{ij}}\left(\vartheta_{i}-\vartheta_{j}\right)+\rho \sum_{i=1}^{N}h_{i}Y_{i}\vartheta_{i}=q^{\prime }+\rho \sum_{i=1}^{N}h_{i}Y_{i}\vartheta_{i}$$

where λ, $\sigma_{b}$, ε, and $R_{u}$ are the mixture thermal conductivity [W m^−1^ K^−1^], the Stefan–Boltzmann constant [W m^−2^ K^−4^], the mixture emissivity [-], and the Universal Gas Constant [J mol^−1^ K^−1^], respectively. Furthermore, $X_{j}$ is the mole fraction of the jth component of the mixture [-], $D^{T}$ is the multicomponent thermal diffusion coefficient for species i [kg m^−1^ s^−1^] [39], $m_{i}$ is the molecular mass of the ith species [kg mol^−1^], $D_{ij}$ is the binary diffusion coefficient of species i and j [m^2^ s^−1^], and $h_{i}$ is the average enthalpy per unit mass associated with the ith species [J kg^−1^]. In Equation (20), the heat flux consists of conduction, radiation, the Dufour effect, and interdiffusion heat fluxes. Thus, the entropy generation occurring in Equation (19) evolves as follows:

(21) $$g_{s}=-\frac{q^{\prime }}{T^{2}}\nabla T+\frac{\Phi }{T}+\frac{1}{T}\sum_{i=1}^{N}\rho Y_{i}\vartheta_{i}\left(f_{i}+\frac{\left(\mu_{c,i}-h_{i}\right)}{T}\nabla T-\nabla \mu_{c,i}\right)-\frac{1}{T}\sum_{i=1}^{N}\mu_{c,i}{\dot{\omega }}_{i}$$

In the context of a mixture of ideal gases, the relationship between the term $\mu_{c,i}-h_{i}$ and the partial specific entropy of species i of the mixture $s_{i}$ can be expressed as ${\mu_{c,i}=h}_{i}-Ts_{i}$. Consequently, Equation (21) can be transformed into the following expression:

(22) $$g_{s}=-\frac{q^{\prime }}{T^{2}}\nabla T+\frac{\Phi }{T}+\frac{1}{T}\sum_{i=1}^{N}\rho Y_{i}\vartheta_{i}\left(f_{i}-s_{i}\nabla T-\nabla \mu_{c,i}\right)-\frac{1}{T}\sum_{i=1}^{N}\mu_{c,i}{\dot{\omega }}_{i}$$

The gradient of chemical potential in Equation (22) is expressed as follows:

(23) $$\nabla \mu_{c,i}=\nabla h_{i}-T\nabla s_{i}-s_{i}\nabla T$$

Substituting Equation (23) into Equation (22) yields the following expressions:

(24) $$g_{s}=-\frac{q^{\prime }}{T^{2}}\nabla T+\frac{\Phi }{T}+\frac{1}{T}\sum_{i=1}^{N}\rho Y_{i}\vartheta_{i}\left(f_{i}-\nabla h_{i}+T\nabla s_{i}\right)-\frac{1}{T}\sum_{i=1}^{N}\mu_{c,i}{\dot{\omega }}_{i}$$

The partial specific entropy for an ideal gas can be calculated using the reference state of entropy as follows:

(25) $$s_{i}=s_{i}^{{}^{\circ}}+\int_{T^{{}^{\circ}}}^{T}\frac{c_{p,i}}{T}dT-R_{i}\ln\frac{p_{i}}{p^{{}^{\circ}}}=s_{i}^{{}^{\circ}}+\int_{T^{{}^{\circ}}}^{T}\frac{c_{p,i}}{T}dT-R_{i}\ln\frac{p}{p^{{}^{\circ}}}-R_{i}\ln X_{i}$$

The specific entropy of the species i [m^2^ s^−2^ K^−1^], which is denoted as $s_{i}^{{}^{\circ}}$, is evaluated at the reference temperature $T^{{}^{\circ}}$[K] and pressure $p^{{}^{\circ}}$ [Pa]. Ri=Rumi is the specific gas constant of the ith species [J kg^−1^ K^−1^], and $p_{i}=X_{i}p$ is the partial pressure of the ith species [Pa]. The specific enthalpy of an ideal gas is calculated as follows:

(26) $$h_{i}=h_{i}^{{}^{\circ}}+\int_{T^{{}^{\circ}}}^{T}c_{p,i}dT$$

Incorporating the ideal gas equation, along with the combination of the specific enthalpy and partial specific entropy, results in the writing of the gradients in the third term on the right-hand side of Equation (24) as follows [40]:

(27) $$-\nabla h_{i}+T\nabla s_{i}=-\frac{1}{\rho_{i}}\left(p\nabla X_{i}+X_{i}\nabla p\right)$$

Substituting Equation (27) into Equation (24) gives the following expression:

(28) $$g_{s}=-\frac{q^{\prime }}{T^{2}}\nabla T+\frac{\Phi }{T}+\frac{1}{T}\sum_{i=1}^{N}\rho Y_{i}\vartheta_{i}\left(f_{i}-\frac{1}{\rho_{i}}\left(p\nabla X_{i}+X_{i}\nabla p\right)\right)-\frac{1}{T}\sum_{i=1}^{N}\mu_{c,i}{\dot{\omega }}_{i}$$

Equation (28) provides a means of computing the rate of entropy generation per unit volume in a mixture of ideal gases within reacting flows. Specifically, $g_{s}$ can be further divided into five distinct generation terms, which are defined as follows:

(29) $$g_{s}={g_{s}}_{h}+{g_{s}}_{m}+{g_{s}}_{c}+{g_{s}}_{\mu }+{g_{s}}_{r}$$

where ${g_{s}}_{h}$, ${g_{s}}_{m}$, ${g_{s}}_{c}$, ${g_{s}}_{\mu }$, and ${g_{s}}_{r}$ are the contributions of heat transfer, mass transfer, heat and mass transfer coupling, viscous dissipation, and reaction, respectively, to in the entropy generation. The coupling between heat and mass transfer is described based on the Dufour and Soret effects. These effects account for the influence of concentration gradients (Soret effect) and temperature gradients (the Dufour effect) on the transfer of heat and mass within the mixture. The velocity of the species depends on both the bulk flow velocity and the diffusion velocity. In a multicomponent mixture, four distinct modes of mass diffusion exist: ordinary diffusion resulting from a concentration gradient, thermal diffusion that is a result of a temperature gradient, pressure diffusion that results from a pressure gradient, and forced diffusion that results from unequal body forces per unit mass among the species. Thus, the diffusion velocity of species i can be defined as follows:

(30) $$\vartheta_{i}=\vartheta_{i,\chi }+\vartheta_{i,T}+\vartheta_{i,p}+\vartheta_{i,f}$$

where the subscripts χ, T, p, and f refer to ordinary, thermal, pressure, and forced diffusion, respectively. However, in a typical combustion system, pressure gradients are not sufficiently large to induce pressure diffusion. Moreover, forced diffusion is usually triggered by charged ions interacting with an electric field, and it is not significant in combustion [37]. Therefore, assuming that the pressure diffusion and body force diffusion are negligible, the diffusion velocity can be written as follows:

(31) $$\vartheta_{i}=\vartheta_{i,\chi }+\vartheta_{i,T}$$

Incorporating Equation (31) into Equation (28) and comparing it to Equation (29) gives the below set of relations for each contribution to the entropy generation rate. The entropy generated due to heat transfer while incorporating conduction and radiation heat transfers can be expressed as follows:

(32) $${g_{s}}_{h}=\frac{1}{T^{2}}\left(\lambda \nabla T-\sigma_{b}\varepsilon T^{4}\right)\nabla T$$

The entropy generated due to mass transfer with respect to body ordinary diffusion velocity corresponding to Equation (27) is given as follows:

(33) $${g_{s}}_{m}=\frac{1}{T}\sum_{i=1}^{N}\rho Y_{i}\vartheta_{i,\chi }\left(f_{i}-\frac{1}{\rho_{i}}\left(p\nabla X_{i}+X_{i}\nabla p\right)\right)$$

The entropy generated due to the coupling effect between mass and heat transfer with respect to thermal diffusion velocity, while accounting for the Dufour effect, can be given as follows:

(34) $${g_{s}}_{c}=\frac{1}{T}\sum_{i=1}^{N}\rho Y_{i}\vartheta_{i,T}\left(f_{i}-\frac{1}{\rho_{i}}\left(p\nabla X_{i}+X_{i}\nabla p\right)\right)-\frac{R_{u}}{T}\sum_{i=1}^{N}\sum_{j=1}^{N}\frac{X_{j}\alpha_{i}}{m_{i}D_{ij}}\left(\vartheta_{i}-\vartheta_{j}\right)\nabla T$$

where the second term on the right-hand side represents the Dufour effect [41]. The entropy generated accounting for viscous dissipation term Φ is given as follows:

(35) $${g_{s}}_{\mu }=\frac{\Phi }{T}$$

and the reaction-related entropy generation is given as follows:

(36) $${g_{s}}_{r}=-\frac{1}{T}\sum_{i=1}^{N}\mu_{c,i}{\dot{\omega }}_{i}$$

Thus, substituting all of the heat fluxes and Equations (18) and (28) into Equation (17) gives the final entropy balance within the CV as follows:

(37) $$\rho \frac{Ds}{Dt}=-\nabla \cdot \left[\frac{1}{T}\left(-\lambda \nabla T+\sigma_{b}\varepsilon T^{4}+R_{u}T\sum_{i=1}^{N}\sum_{j=1}^{N}\frac{X_{j}D^{T}}{m_{i}D_{ij}}\left(\vartheta_{i}-\vartheta_{j}\right)+\rho \sum_{i=1}^{N}h_{i}Y_{i}\vartheta_{i}-\sum_{i=1}^{N}\left(\mu_{c,i}\rho Y_{i}\vartheta_{i}\right)\right)\right]\;-\frac{1}{T^{2}}\left[-\lambda \nabla T+\sigma_{b}\varepsilon T^{4}+R_{u}T\sum_{i=1}^{N}\sum_{j=1}^{N}\frac{X_{j}D^{T}}{m_{i}D_{ij}}\left(\vartheta_{i}-\vartheta_{j}\right)\right]\nabla T+\frac{\Phi }{T}\;+\frac{1}{T}\sum_{i=1}^{N}\rho Y_{i}\vartheta_{i}\left(f_{i}-\frac{1}{\rho_{i}}\left(p\nabla X_{i}+X_{i}\nabla p\right)\right)-\frac{1}{T}\sum_{i=1}^{N}\mu_{c,i}{\dot{\omega }}_{i}$$

Assuming that the Dufour effect is negligible, the pressure gradient is also negligible, there are no external body forces, and viscous dissipation is neglected when the Mach number is small. Thus, Equation (37) reduces to the following expression:

(38) $$\rho \frac{Ds}{Dt}=-\nabla \cdot \left[\frac{1}{T}\left(-\lambda \nabla T+\sigma_{b}\varepsilon T^{4}+\rho \sum_{i=1}^{N}h_{i}Y_{i}\vartheta_{i}-\sum_{i=1}^{N}\left(\mu_{c,i}\rho Y_{i}\vartheta_{i}\right)\right)\right]-\frac{1}{T^{2}}\left[-\lambda \nabla T+\sigma_{b}\varepsilon T^{4}\right]\nabla T-\rho \frac{R_{u}}{\bar{m}}\sum_{i=1}^{N}\vartheta_{i}\nabla X_{i}-\frac{1}{T}\sum_{i=1}^{N}\mu_{c,i}{\dot{\omega }}_{i}$$

where $\bar{m}$ is the average molecular mass of the mixture and is defined as follows:

(39) $$\bar{m}=\sum_{i=1}^{N}X_{i}m_{i}$$

Using the definitions of gradient, divergence, and Laplacian operators in spherical coordinates, Equation (38) can be translated into the spherical coordinates as follows:

(40) $$\begin{array}{ll} \rho (\frac{\partial s}{\partial t}+V_{r}\frac{\partial s}{\partial r}+\frac{V_{\theta }}{r}\frac{\partial s}{\partial \theta } & +\frac{V_{\varphi }}{r\sin\theta }\frac{\partial s}{\partial \varphi })\\ & =\left[\frac{1}{r^{2}}\frac{\partial }{\partial r}\left(\frac{\lambda }{T}r^{2}\frac{\partial T}{\partial r}\right)+\frac{1}{r^{2}\sin\theta }\frac{\partial }{\partial \theta }\left(\frac{\lambda }{T}\sin\theta \frac{\partial T}{\partial \theta }\right)+\frac{1}{r^{2}\sin^{2}\theta }\frac{\partial }{\partial \varphi }\left(\frac{\lambda }{T}\frac{\partial T}{\partial \varphi }\right)\right]\\ & -\left(\frac{1}{r^{2}}\frac{\partial }{\partial r}\left(r^{2}\frac{q_{R}}{T}\right)+\frac{1}{r\sin\theta }\frac{\partial }{\partial \theta }\left(\sin\theta \frac{q_{R}}{T}\right)+\frac{1}{r\sin\theta }\frac{\partial }{\partial \varphi }\left(\frac{q_{R}}{T}\right)\right)\\ & -\left[\frac{1}{r^{2}}\frac{\partial }{\partial r}\left(r^{2}\rho \sum_{i=1}^{N}c_{pi}Y_{i}\vartheta_{i,r}\right)+\frac{1}{r\sin\theta }\frac{\partial }{\partial \theta }\left(\rho \sin\theta \sum_{i=1}^{N}c_{pi}Y_{i}\vartheta_{i,\theta }\right)+\frac{1}{r\sin\theta }\frac{\partial }{\partial \varphi }\left(\rho \sum_{i=1}^{N}c_{pi}Y_{i}\vartheta_{i,\varphi }\right)\right]\\ & -\left[\frac{1}{r^{2}}\frac{\partial }{\partial r}\left(r^{2}\frac{\rho }{T}\sum_{i=1}^{N}\mu_{c,i}Y_{i}\vartheta_{i,r}\right)+\frac{1}{r\sin\theta }\frac{\partial }{\partial \theta }\left(\frac{\rho }{T}\sin\theta \sum_{i=1}^{N}\mu_{c,i}Y_{i}\vartheta_{i,\theta }\right)+\frac{1}{r\sin\theta }\frac{\partial }{\partial \varphi }\left(\frac{\rho }{T}\sum_{i=1}^{N}\mu_{c,i}Y_{i}\vartheta_{i,\varphi }\right)\right]\\ & +\frac{\lambda }{T^{2}}{\left(\frac{\partial T}{\partial r}+\frac{1}{r}\frac{\partial T}{\partial \theta }+\frac{1}{r\sin\theta }\frac{\partial T}{\partial \varphi }\right)}^{2}-\frac{q_{R}}{T^{2}}\left(\frac{\partial T}{\partial r}+\frac{1}{r}\frac{\partial T}{\partial \theta }+\frac{1}{r\sin\theta }\frac{\partial T}{\partial \varphi }\right)-\rho \frac{R_{u}}{\bar{m}}\sum_{i=1}^{N}\left(\vartheta_{i,r}\frac{\partial X_{i}}{\partial r}+\vartheta_{i,\theta }\frac{1}{r}\frac{\partial X_{i}}{\partial \theta }+\frac{\vartheta_{i,\varphi }}{r\sin\theta }\frac{\partial X_{i}}{\partial \varphi }\right)\\ & -\frac{1}{T}\sum_{i=1}^{N}\mu_{c,i}{\dot{\omega }}_{i} \end{array}$$

where $c_{pi}$ is the constant pressure specific heat of the species i [J kg^−1^ K^−1^], and $q_{R}$ is the radiation heat flux. In Equation (40), r, θ, and φ represent radial distance, polar angle, and azimuthal angle of an arbitrary point in space using spherical coordinates. For the detailed conversion steps of cartesian coordinates into spherical coordinates and the vector calculus identified using spherical coordinates, we referred to books by Hassani [42] and Zwillinger [43]. Overall, Equation (40) can be used to find the entropy change in a reacting flow in spherical coordinates, such as droplet evaporation, and we conduct these experiments in the below sections.

## 3. Droplet Evaporation Model (d^2^ Law)

**Figure 2:**
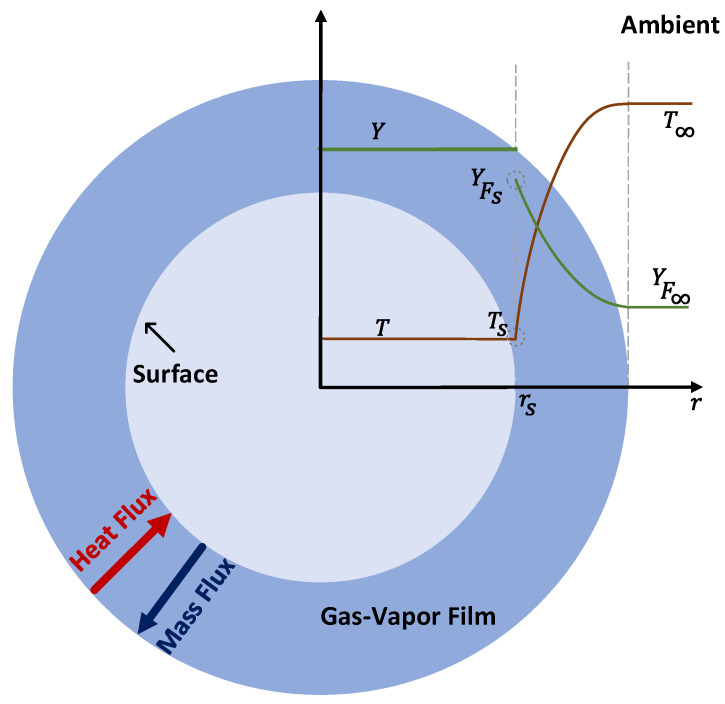
Schematic representation of an evaporating droplet surrounded by gas with mass fractions and temperature distributions along the radial direction.

To test this model, an isolated, single-component, liquid, and spherical droplet is suddenly exposed to an infinite hot gaseous medium charged with nitrogen, and it starts to vaporize due to the system being spherically symmetric, as indicated in Figure 2. Since the liquid density is significantly larger than that of the ambient gas, the gas phase is assumed to be quasi-steady, and gas-phase heat and mass transfers are assumed to be larger than the corresponding heat and mass transfers in the liquid phase. Thus, the transient heating of the liquid takes longer than that of the gas phase; subsequently, the internal circulation within the liquid droplet is neglected, and the droplet temperature is assumed to be uniform and constant. As a result, the corresponding d^2^ model is essentially a steady-state gas-phase model, with the liquid-phase heat and mass transport processes being neglected. The gaseous mixture is assumed to be ideal, and the Soret and Dufour effects are ignored. The droplet has zero relative velocity with the ambient gas, the evaporation process is isobaric, and there is no effect of other droplets and external forces. Moreover, the saturation vapor pressure is assumed to be located at the droplet surface, as well as at the thermodynamic equilibrium between the two phases.

Using the continuity equation for the gas-phase mixture while incorporating spherical symmetry to remove the dependency on angular coordinates results in the following expression:

(41) $$\frac{1}{r^{2}}\frac{\partial \left(\rho V_{r}r^{2}\right)}{\partial r}=0$$

which can be integrated to recover

(42) $$\rho V_{r}r^{2}\;=\;\mathrm{constant}$$

Initially, the gas phase has no velocity, as no evaporation is happening. Once evaporation starts, mass will be added to the gas phase, with the boundary moving inwards at the velocity of the gas ($V_{r}$). The mass will be added to the gas phase, which in the form of a mass flow rate equals

(43) $$\dot{m}=4\pi \rho V_{r}r^{2}$$

where $\dot{m}$ is the droplet mass vaporization rate [kg s^−1^], and $4\pi r^{2}$ is the area of the droplet surface. Similar assumptions of quasi-steady flow and spherical symmetry can be made to reduce the species conservation equation to the following expression:

(44) $$\rho r^{2}V_{r}\frac{\partial Y_{i}}{\partial r}+\frac{\partial \left({\rho r}^{2}Y_{i}\vartheta_{i,r}\right)}{\partial r}=0$$

Incorporating the diffusion flux while assuming only Fickian mass diffusion and substituting this component in Equation (44) yields the following expression:

(45) $$\rho r^{2}V_{r}\frac{\partial Y_{i}}{\partial r}=\frac{\partial }{\partial r}\left(\rho D_{i}r^{2}\frac{\partial Y_{i}}{\partial r}\right)$$

Integrating Equation (45) while incorporating Equation (43) gives the following expression:

(46) $$\frac{\dot{m}}{4\pi }Y_{i}\left(r\right)+C_{1}=\rho D_{i}r^{2}\frac{\partial Y_{i}}{\partial r}$$

where $C_{1}$ is the constant that results from the integration. To find this constant, a boundary condition is required for the mass balance at the droplet interface. Advection and diffusion on the gas side are equal to advection on the liquid side, as shown in the following equation:

(47) $$\frac{\dot{m}}{4\pi }{Y_{i}}_{s}-{\left.\rho D_{i}r^{2}\frac{\partial Y_{i}}{\partial r}\right|}_{s}=\frac{\dot{m}}{4\pi }{\delta_{i}}_{F}$$

where subscripts F and s denote fuel species and the surface of the droplet, respectively. Moreover, $\delta_{i}$ is the delta function for each species. For non-vaporizing species, radial advection and diffusion cancel each other. The right-hand side of Equation (47) is not zero for only the vaporizing species (i.e., $\delta_{i}=1$). Here, it is important to note that the evaporation rate ($\dot{m}$) is equivalent for both the liquid and gas phases. Thus, the left-hand side of Equation (47) describes the mass gained by the gas, and the right-hand side of the equation describes the mass lost by the liquid. By comparing Equations (46) and (47), $C_{1}$ is shown to be equal to $-\frac{\dot{m}}{4\pi }$, and Equation (46) results in the following expression:

(48) $$\frac{\dot{m}}{4\pi }\left(Y_{i}\left(r\right)-1\right)=\rho D_{i}r^{2}\frac{\partial Y_{i}}{\partial r}$$

Integrating Equation (48) from the droplet surface to the ambient requires us to define the following boundary conditions for species mass fractions at the droplet surface ($r_{s}$) and the ambient (∞):

(49) $$r=r_{s};\;Y_{F}={Y_{F}}_{s}$$

(50) $$r=\infty ;Y_{F}={Y_{F}}_{\infty }$$

Thus, performing a separation of variables and integrating Equation (48) at the droplet surface to the ambient results in the following expression:

(51) $$\dot{m}=2\pi d_{s}\rho D_{i}\ln\left(\frac{{Y_{F}}_{\infty }-1}{{Y_{F}}_{s}-1}\right)$$

where $d_{s}$ is the droplet diameter. Subsequently, we define the Spalding mass transfer number as follows:

(52) $$B_{M}=\frac{{Y_{F}}_{\infty }-{Y_{F}}_{s}}{{Y_{F}}_{s}-1}$$

and incorporating Equation (52) into Equation (51) gives the following expression:

(53) $$\dot{m}=2\pi d_{s}\rho D_{i}\ln\left(1+B_{M}\right)$$

At the droplet surface, equating the droplet mass vaporization rate with the droplet mass consumption rate yields the following expression:

(54) $$\dot{m}=-\frac{d}{dt}\left(\frac{1}{6}\pi d_{s}^{3}\rho_{l}\right)$$

where the subscript l represents liquid phase. We then define the evaporation rate constant as follows:

(55) $$K=-\frac{8\rho D_{i}}{\rho_{l}}\ln\left(1+B_{M}\right)$$

and incorporating Equation (53) into Equation (54) gives the following expression:

(56) $$\frac{d}{dt}\left(d_{s}^{2}\right)=K$$

It is evident that this evaporation parameter linearly depends on the transport properties through $\rho D_{i}$. Next, integrating Equation (56) leads to the following expression:

(57) $$d_{s}^{2}=d_{s0}^{2}+Kt$$

where $d_{s0}$ is the initial droplet diameter. Equation (57) is the basis of the d^2^ law in droplet evaporation, stating that the square of the droplet diameter linearly decreases with time as vaporization occurs.

An analogous approach can be conducted to solve the reduced energy conservation equation. The energy conservation equation that uses the prior assumptions, as well as quasi-steadiness of the gas phase and spherical symmetry, results in the following expression:

(58) $$\rho c_{p}V_{r}\frac{\partial T}{\partial r}=\left[\frac{1}{r^{2}}\frac{\partial }{\partial r}\left(\lambda r^{2}\frac{\partial T}{\partial r}\right)\right]-\left[\frac{1}{r^{2}}\frac{\partial }{\partial r}\left(r^{2}\rho T\sum_{i=1}^{N}c_{pi}Y_{i}\vartheta_{i,r}\right)\right]$$

Retaining ordinary diffusion flux only with respect to Equation (31), Fick’s law of diffusion gives radial diffusion velocity as follows:

(59) $$\vartheta_{i,r}=-\frac{D_{i}}{Y_{i}}\frac{\partial Y_{i}}{\partial r}$$

and all binary diffusion coefficients for each pair of species in the mixture are equal. Moreover,

(60) $$\frac{\partial }{\partial r}\left(\sum_{i=1}^{N}h_{i}Y_{i}\right)=\sum_{i=1}^{N}h_{i}\frac{\partial Y_{i}}{\partial r}+\sum_{i=1}^{N}Y_{i}\frac{\partial h_{i}}{\partial r}$$

Substituting Equations (59) and (60) into Equation (58) results in the following expression:

(61) $$\rho V_{r}r^{2}c_{p}\frac{\partial T}{\partial r}=\frac{\partial }{\partial r}\left(\lambda r^{2}\frac{\partial T}{\partial r}\right)$$

This equation is generally a representation of the Shvab–Zeldovich formulation for conservation equations in reacting flows, which assumes that ρD is constant as an acceptable approximation, and this quantity can be replaced by $\frac{\lambda }{c_{p}}$, since the Lewis number is assumed to be unity. Furthermore, an average specific heat for all species present in the gaseous mixture is more relevant in this formulation, highlighting the significance of Lewis number unity [41,44]. The thermal properties of the gaseous mixture in this study are assumed to be constant and have been calculated as mixture-averaged properties, with respect to the droplet surface mass fractions and ambient temperatures. Integrating Equation (61) while incorporating Equation (43) gives the following expression:

(62) $$\frac{\dot{m}}{4\pi }c_{p}\left(T-T_{s}\right)+C_{2}=\lambda r^{2}\frac{\partial T}{\partial r}$$

where $C_{2}$ is the constant that results from the integration. To find this constant, a boundary condition is required to determine the energy balance at the droplet interface. This boundary condition includes the gas-phase conductive flux, convective flux to the droplet surface, and the energy required to vaporizing the liquid at the surface, as shown in the following expression:

(63) $${\left.\lambda r^{2}\frac{\partial T}{\partial r}\right|}_{s}=\frac{{\dot{q}}_{l}}{4\pi }+\frac{\dot{m}}{4\pi }L=\frac{\dot{m}}{4\pi }c_{p}\left(T-T_{s}\right)+\frac{\dot{m}}{4\pi }L$$

where ${\dot{q}}_{l}$ is the convective heat flux moving inward at the droplet surface [J s^−1^], and L is the latent heat of vaporization [J kg^−1^]. Comparing Equations (62) and (63), $C_{2}$ is equal to $\frac{\dot{m}}{4\pi }L$, and Equation (62) yields the following expression:

(64) $$\frac{\dot{m}c_{p}}{4\pi }\left(T-T_{s}+\frac{L}{c_{p}}\right)=\lambda r^{2}\frac{\partial T}{\partial r}$$

Integrating Equation (64) from the droplet surface to the ambient requires that we define the following boundary conditions at the droplet surface and the ambient:

(65) $$r=r_{s};T=T_{s}$$

(66) $$r=\infty ;T=T_{\infty }$$

Thus, integrating Equation (64) results in the following expression:

(67) $$\dot{m}=2\pi d\frac{\lambda }{c_{p}}\ln\left(\frac{c_{p}\left(T_{\infty }-T_{s}\right)+L}{L}\right)$$

Subsequently, we define the Spalding heat transfer number as follows:

(68) $$B_{T}=\frac{c_{p}\left(T_{\infty }-T_{s}\right)}{L}$$

and incorporating Equation (68) into Equation (67) gives the following expression:

(69) $$\dot{m}=2\pi d\frac{\lambda }{c_{p}}\ln\left(1+B_{T}\right)$$

Comparing Equation (53) with Equation (69) finds the following expression:

(70) $$2\pi d\rho D_{i}\ln\left(1+B_{M}\right)=2\pi d\frac{\lambda }{c_{p}}\ln\left(1+B_{T}\right)$$

Thus, in the case when Le=1

(71) $$B_{M}=\frac{{Y_{F}}_{\infty }-{Y_{F}}_{s}}{{Y_{F}}_{s}-1}=B_{T}=\frac{c_{p}\left(T_{\infty }-T_{s}\right)}{L}$$

As a result, Equation (71) relates two unknowns—${Y_{F}}_{s}$ and $T_{s}$—for which one additional equation is needed to generate a solution. According to Dalton’s law for ideal gases, the partial pressures of chemical species are a function of mole fractions. Thus, the partial pressure of the fuel at the surface ($p_{Fs}$) can be related to the pressure of the system (p) and the mole fraction of the fuel at the surface ($X_{Fs}$) as follows:

(72) $$p_{Fs}=pX_{Fs}$$

In a medium that only uses fuel and nitrogen as its components, the mole fraction of the fuel can be written as follows:

(73) $$X_{Fs}=\frac{\frac{Y_{Fs}}{M_{F}}}{\frac{Y_{Fs}}{M_{F}}+\frac{Y_{N_{2}s}}{M_{N_{2}}}}$$

Thus, the partial pressure of the fuel as a function of mass fractions at the droplet surface can be obtained from the following equation:

(74) $$p_{Fs}=\frac{\frac{Y_{Fs}}{M_{F}}}{\frac{Y_{Fs}}{M_{F}}+\frac{1-Y_{Fs}}{M_{N_{2}}}}p$$

Subsequently, the additional equation can be found using the Clausius–Clapeyron phase equilibrium relation at the droplet surface as follows:

(75) $$\ln\frac{p_{Fs}}{p_{F,\;ref}}=\frac{L}{R}\left(\frac{1}{T_{F,\;ref}}-\frac{1}{T_{s}}\right)$$

where $T_{F,\;ref}$ represents the boiling temperature of the liquid fuel, and $p_{F,\;ref}$ represents the saturation pressure at the boiling temperature that can be calculated using the following Antoine equation:

(76) $$\log\left(p_{F,\;ref}\right)=A-\left(\frac{B}{T_{F,\;ref}+C}\right)$$

where A, B, and C are constants specific to the liquid fuel. As a result, after substituting Equation (74) into Equation (75), there are three equations and three unknowns; thus, the system can fully be solved. In other words, the analytical d^2^ law model consists of solving the conservation of species through Equation (45), the conservation of energy through Equation (61), and equilibrium at the interface through Equation (75) to find the unknowns of *Y*, *T*, and $\dot{m}$.

## 4. Numerical Model

In order to incorporate the entropy equation, a numerical model is proposed to solve the chemical species and energy transport equations (Equations (45) and (61)), as well as pertinent boundary conditions for the gas-phase medium at the droplet interface and ambient (Equations (49) and (50) and Equations (65) and (66)), after considering the same assumptions made for the d^2^ law. Therefore, the entropy balance of Equation (40) will be reduced to the following expression:

(77) $$\rho V_{r}\frac{\partial s}{\partial r}=\left[\frac{1}{r^{2}}\frac{\partial }{\partial r}\left(\frac{\lambda }{T}r^{2}\frac{\partial T}{\partial r}\right)\right]-\left[\frac{1}{r^{2}}\frac{\partial }{\partial r}\left(r^{2}\frac{\rho }{T}\sum_{i=1}^{N}\mu_{c,i}Y_{i}\vartheta_{i,r}\right)\right]+\frac{\lambda }{T^{2}}{\left(\frac{\partial T}{\partial r}\right)}^{2}-\rho \frac{R_{u}}{\bar{m}}\sum_{i=1}^{N}\left(\vartheta_{i,r}\frac{\partial X_{i}}{\partial r}\right)$$

In Equation (77), the left-hand side represents the advection of entropy, and the right-hand side terms represent the conduction of entropy, diffusion of entropy, entropy generated due to conduction, and entropy generated due to mass transfer. Thus, the volumetric rate of entropy generation yields the following equation:

(78) $$g_{s}=\frac{\lambda }{T^{2}}{\left(\frac{\partial T}{\partial r}\right)}^{2}-\rho \frac{R_{u}}{\bar{m}}\sum_{i=1}^{N}\left(\vartheta_{i,r}\frac{\partial X_{i}}{\partial r}\right)$$

The governing equations were discretized using a finite difference method in which the advection terms employ a first-order upwind scheme, and diffusion terms were treated based on the second order central difference using an overall convergence criterion of 10^−8^ for both the chemical species and the temperature. Grid independence was found at a grid size of 7.96 µm when increasing the number of grids was found to cause negligible difference (maximum 0.1%). The gas-phase domain included 200 times the droplet diameter from the droplet surface to the ambient, which was similar to measures used in other studies outlined in the literature [23,45].

## 5. Model Validation

**Figure 3:**
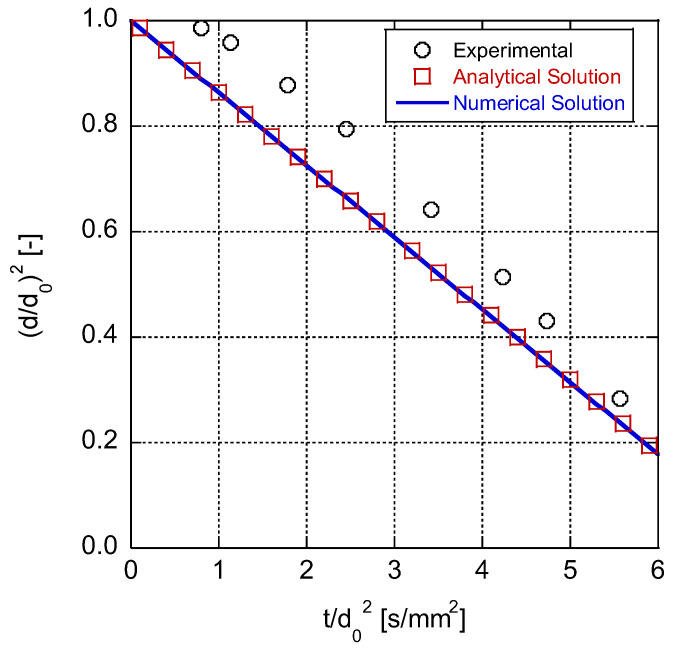
Variations in squared non-dimensional droplet diameter over time; comparison between analytical solution and numerical model and experimental data [21].

The results from the model were compared to those of the work of Nomura et al. [21]. They experimentally investigated the evaporation of an n-heptane droplet in a wide range of ambient temperatures and pressures within a microgravity environment. Figure 3 illustrates the results from the model for a 0.8-millimeter n-heptane droplet after exposure to an environment charged with nitrogen at 471 K and 0.1 MPa. The omission of the transient heating of the liquid droplet from its initial temperature to its vaporization point in the current model caused the droplet to evaporate at an accelerated rate compared to those of the experimental data before it reached the evaporation temperature in reality. This disparity was more pronounced when dealing with heavier fuels, which have higher boiling points and require more time to convert into the vapor phase. Moreover, the fuel droplet in the experiment was suspended from the tip of a quartz fiber so that it could be easily controlled and imaged. In reality, the fiber conducted heat to the droplet since it had a greater thermal conductivity than the gas [46], which caused a phenomenon that was not included in the model. Moreover, the model assumed constant thermophysical properties for the gas phase, while in reality, they changed with temperature and composition. Here, since the flow velocity was relatively small, the assumption of dynamic incompressibility can be made (Mach number << 1), and the gas density can be considered constant [47]. This assumption has been also utilized by Wu et al. [48] and Renksizbulut and Yuen [49]. Given that the model’s results (to be discussed) exhibit relatively acceptable agreement with the experimental findings, this result indicates that this simplifying assumption allows the model to capture the dominant physical processes governing droplet evaporation. Future work could compare the results of a compressible flow simulation to those of the dynamically incompressible flow solutions to highlight the influence of compressibility on the findings.

Although the d^2^ law model may not be able to make precise predictions of droplet evaporation behavior, it can serve as a beneficial tool for grasping the core principles of heat and mass transfer involved in this process. The model’s simplicity in mathematical formulations makes it particularly useful for introducing new researchers to the field and facilitating their understanding of entropy changes during droplet evaporation. Consequently, the model has been chosen as an approachable means of investigating such phenomena.

## 6. Results and Discussion

It has been observed that during the combustion of alkanes, a cool flame is first formed at lower temperatures, which is followed by a high temperature ignition process. Thus, as a starting point, this study focuses on n-heptane droplet evaporation. N-heptane is selected as abundant experimental data are available regarding its evaporation and ignition, it is considered to be a primary reference fuel used for octane rating, it is a surrogate for the n-alkane family in more complex fuel blends, and it encounters the LTC regime when igniting under certain conditions [25,50]. Table 1 includes the parameters and values used in the model.

**Figure 4:**
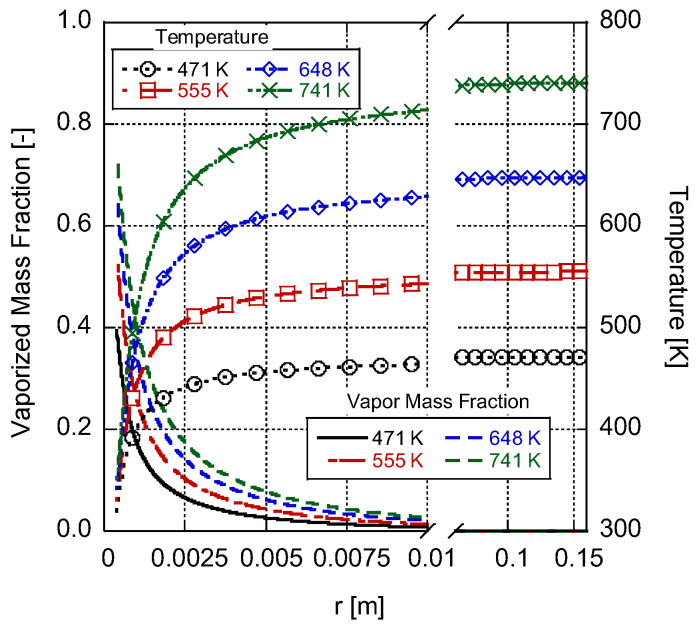
Gas-phase temperature and vaporized fuel mass fraction change over radial distance at a 0.1-megapascal ambient pressure for a 0.8-millimeter n-heptane droplet in different ambient temperatures.

Figure 4 presents the variations in gas-phase temperature and vaporized fuel mass fraction as functions of distance from the droplet surface while considering different ambient temperatures under a constant pressure of 0.1 MPa. As illustrated and expected, an increase in ambient temperature leads to a higher droplet evaporation rate through the growth of the Spalding heat transfer number in Equation (68). This effect arises due to the larger temperature gradient between the droplet surface and the ambient, facilitating diffusive heat transfer towards the droplet. It is important to note that the model assumes a steady-state condition, meaning that the temperature at the droplet surface also increases in response to the increased ambient temperature. This elevated droplet surface temperature further enhances the evaporation process, resulting in a greater vaporized fuel mass fraction at both the droplet surface and as we move further away from the droplet.

**Figure 5:**
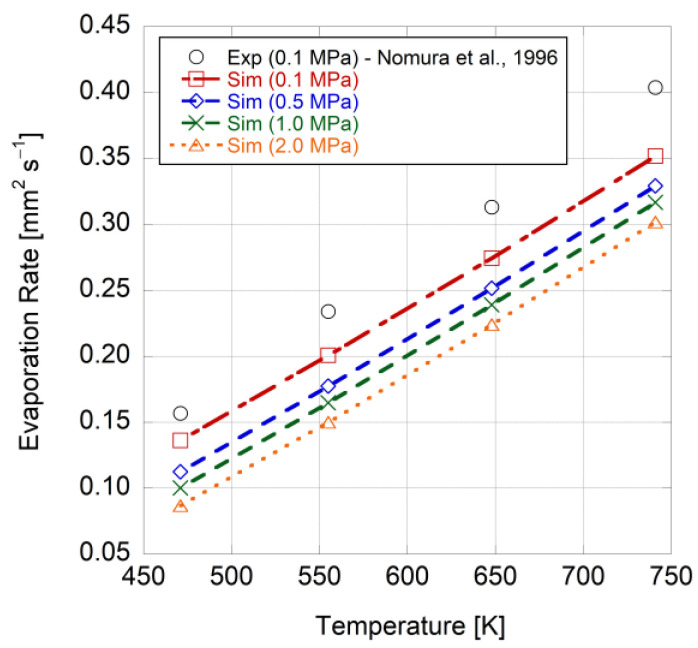
Vaporization rate of an n-heptane droplet based on the ambient temperature for different ambient pressures in microgravity [21].

Figure 5 illustrates the vaporization rate of an n-heptane droplet in microgravity under various ambient temperatures and pressures, as predicted via the model and observed in the experimental work (a 0.1-megapascal pressure) conducted by Nomura et al. [21]. The evaporation rate is calculated using Equation (52) with respect to Spalding mass and heat transfer numbers (Equations (52) and (68)), which are functions of the droplet mass vaporization rate (Equations (53) and (69)). The model generally underestimates the evaporation rate compared to the experimental results, indicating the significance of heat transfer from the support fiber used in the experiment, as well as transient heating and internal circulation within the droplet. Here, an increase in the ambient pressure leads to a decrease in the evaporation rate. This effect occurs as higher pressures impede evaporation by raising the boiling temperature. As a result, the droplet surface requires a higher vapor pressure to overcome the elevated boiling temperature. Furthermore, when comparing droplet evaporation at the same pressure, the evaporation rate increases with higher ambient temperatures. The higher temperatures provide fuel molecules with greater kinetic energy, enabling them to surpass the intermolecular forces that keep them in the liquid phase. Consequently, more molecules reach the droplet surface and evaporate, resulting in an elevated evaporation rate.

**Figure 6:**
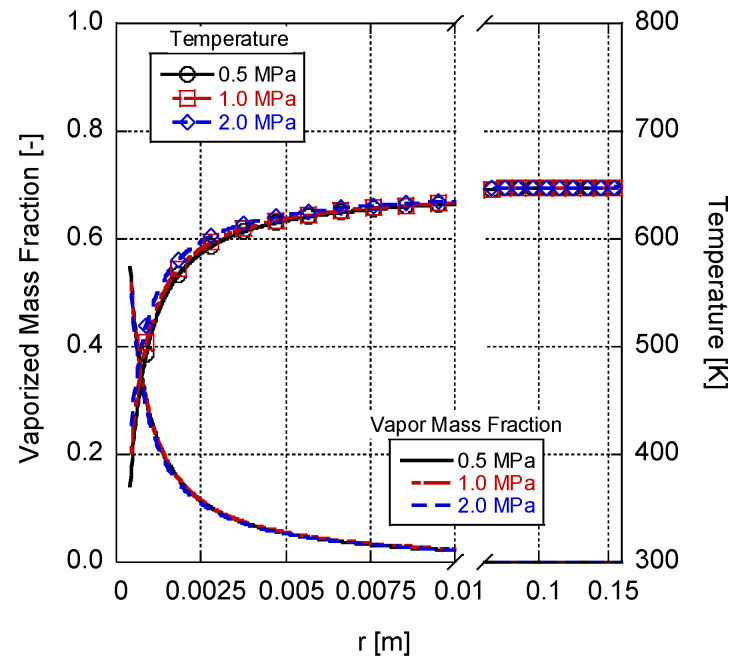
Gas-phase temperature and vaporized fuel mass fraction changes over radial distance at a 648-kelvin ambient temperature for a 0.8-millimeter n-heptane droplet in different ambient pressures.

In Figure 6, the variations in the gas-phase temperature and vaporized fuel mass fraction are depicted as functions of distance from the droplet surface for different ambient pressures while maintaining a constant ambient temperature of 648 K. The chosen ranges of temperatures and pressures promote the low-temperature combustion of n-heptane [25]. As mentioned earlier, the growth in ambient pressure results in a reduction in the vaporized fuel mass fraction, both at the droplet surface and throughout the gas-phase domain. However, it is worth noting that this decrease is relatively small compared to the variations caused by different ambient temperatures. This outcome happens since the Spalding mass and heat transfer numbers are not directly influenced by the ambient pressure. Instead, the effect of pressure is observed in the Clausius–Clapeyron equation through the vapor pressure of the fuel. Furthermore, a slight increase in the gas-phase temperature profile can be observed based on the growth of the ambient pressure. This result can be attributed to higher droplet surface temperatures that occur under elevated pressure conditions, which contribute to a corresponding increase in the surrounding gas-phase temperature.

**Figure 7:**
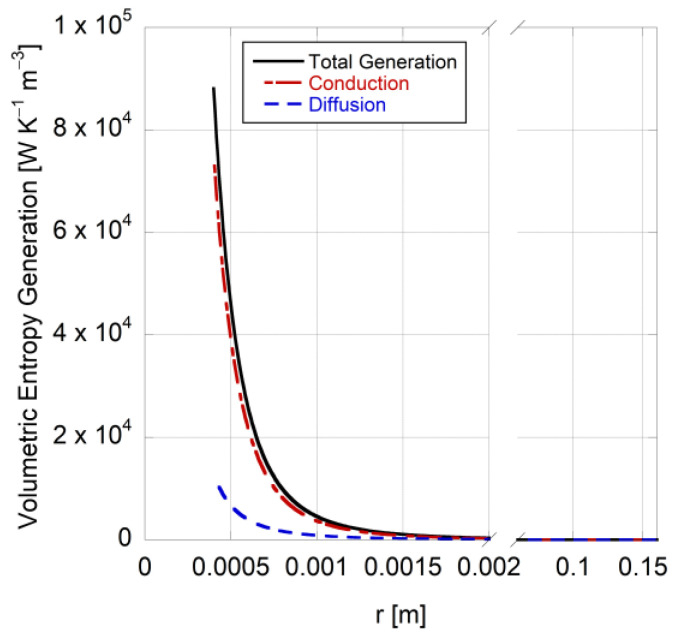
Entropy generation due to each irreversible process involved in a 0.8-millimeter n-heptane droplet evaporation at 648 K and 0.1 MPa.

Figure 7 provides the volumetric entropy generation rate for irreversible heat conduction and mass diffusion processes as a function of distance from the droplet surface, as described in Equation (78), with the total entropy generation denoted as the sum of these two processes. At the droplet surface, the entropy generation rate is highest due to the presence of the largest temperature gradient (see Figure 4), which leads to significant entropy generation through conduction heat transfer. The entropy flow due to mass diffusion is less important, accounting for only 14.4% of the total entropy generation rate. The entropy generation due to viscous dissipation is neglected in this study in order to simplify the model maintaining the spherical symmetry, which was an assumption shown to be correct [54]. Incorporating droplet combustion into the model will add the reaction term in Equation (40) to Equation (78), which could substantially change the total entropy generation rate. Chemical reactions will introduce additional sources of entropy generation, such as energy dissipation during chemical transformation or changes in the species distribution within the gaseous mixture. Thus, the entropy generated due to reactions happening in the medium will likely be the major contributor to the total entropy generation, since it accompanies the formation and decomposition of reactive intermediates, as well as the redistribution of energy and molecular configurations, along with influencing the temperature gradient. In the case of LTC, where the combustion temperature is lower than traditional high-temperature combustion, the reaction-driven entropy generation becomes particularly important. The reduced flame temperatures and longer residence times create conditions that favor radical-based reactions, autoignition processes, or multi-step chain reactions. These reactions can be significantly exothermic in nature and result in substantial entropy generation due to the release of energy and the formation of products with higher configurational randomness. Such analysis also provides information about the extent to which thermal energy is converted into usable work in a second law framework and identifies possible optimization pathways in LTC systems.

**Figure 8:**
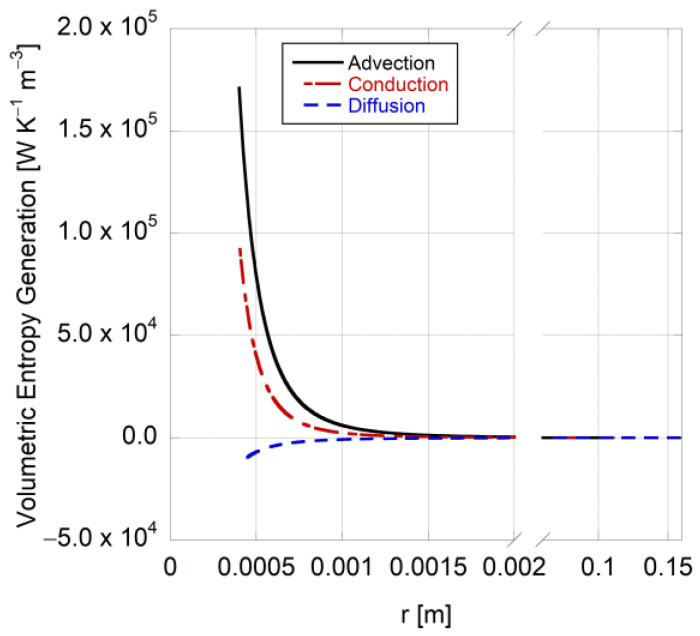
Entropy flow due to each reversible process involved in a 0.8-millimeter n-heptane droplet evaporation at 648 K and 0.1 MPa.

Figure 8 shows the volumetric flow of the entropy due to the reversible processes of advection (i.e., the flow of entropy caused by the vaporized gas velocity away from the droplet), conduction (i.e., the flow of entropy caused by heat conduction), and diffusion (the flow of entropy caused by mass diffusion). Interestingly, entropy flow due to mass diffusion is computed as negative and significantly smaller than those of the other two components. The diffusion of vaporized fuel molecules is outward, causing molecules to move away from the droplet surface. In fact, diffusion of molecules tends to homogenize the concentration distribution, reducing the local concentration gradients and the disorder within the system. As a result, this process leads to a decrease in entropy and contributes negatively to Equation (77). This negative value for entropy diffusion is also reported by Nishida et al. [54] for pre-mixed flames. Notably, mass transfer is minimized in ambient conditions, highlighting minimum entropy generation at equilibrium with the ambient. The entropy flow due to advection (Stefan velocity) and conduction (temperature gradient) are approximately of the same order of magnitude, with advection being 70% more than that of conduction. This outcome can be attributed to the fact that the conductive flow of the entropy is generally limited to the immediate vicinity of the heat transfer region and tends to locally redistribute entropy without significantly affecting the broader entropy profile, in spite of it being the second contributor to the entropy change within the medium.

## 7. Conclusions

Low-temperature combustion (LTC) is an advanced control strategy used in internal combustion engines that holds great promise in mitigating the detrimental effects of particulate matter and nitrogen oxide emissions. In order to fully comprehend LTC and expand its operational range, it is crucial to delve into the simulation and analysis of cool flame behavior and the negative temperature coefficient (NTC) regime. Understanding the mechanisms and dynamics of cool flames can help to optimize LTC strategies. Additionally, the NTC regime, which is characterized by a decrease in the ignition delay with decreasing temperature, has a significant influence on combustion timing and efficiency. By utilizing microgravity droplet combustion experiments in conjunction with a comprehensive model that incorporates the conservation of entropy, a better understanding of the underlying physics and chemistry of these processes can be achieved. This knowledge not only facilitates the identification and characterization of LTC behavior, but also contributes to enhancing overall efficiency through the analysis of availabilities and irreversibilities. 

This effort provides a comprehensive and detailed derivation of the entropy balance equation in reacting flows, presenting a unified framework that encompasses all pertinent information. It elucidates the impact of various transport phenomena on total entropy generation. The derived balance equation incorporates contributions from entropy flow that result from advection, conduction, and diffusion, as well as entropy generation, due to heat and mass transfer, the coupling effect between heat and mass transfer, the influence of chemical reactions, viscous dissipation, pressure effects, and external forces. This study begins by introducing a mathematical model that serves as a foundation for our analysis. Our model focuses on the evaporation of fuel droplets in a high-temperature and microgravity environment, employing the d^2^ law. To verify this model’s accuracy, its predictions are compared to and validated against experimental results. Moreover, the identified flaws of the model are discussed, highlighting areas where the model could be enhanced and refined. These discussions serve as a basis for proposing potential pathways and strategies to improve the existing model. In the subsequent sections, a numerical model is introduced that incorporates the second law of thermodynamics and considerations for entropy generation. The effects of ambient temperature and pressure on the temperature, vaporized fuel mass fraction, and evaporation rate of n-heptane droplets are investigated. Two major contributors to entropy generation within the gaseous mixture, namely conduction and diffusion, are discussed, and the mechanisms through which entropy is transferred are characterized. Future research directions should focus on incorporating droplet combustion into the model to investigate the relationships between reactions, entropy generation, energy dissipation, and the identification of LTC pathways and kinetics. This investigation would provide valuable information regarding the interplay between chemical transformations, entropy change, and energy losses, aiding in the optimization of LTC strategies and the development of more efficient combustion systems.

## Figures and Tables

**Table 1:** N-heptane properties and other parameters used in the model.

**Parameter**	**Value**	**Reference**
Liquid density [kg m^−3^]	684	[51]
Fuel vaporization enthalpy [J kg^−1^]	359,245.5	[52]
Fuel boiling temperature [K]	371.5	[52]
A, B, and C (Antione equation constants)	4.02832, 1268.636, −56.199	[53]
Droplet diameter [m]	0.0008	[21]

## Data Availability

The data and software program used in this study are openly available at https://github.com/depcik/droplet-evaporation-model, accessed on 13 August 2023.

## Abbreviations

CV	Control volume
HCCI	Homogeneous charge compression ignition
LTC	Low-temperature combustion
NTC	Negative temperature coefficient
NOx	Nitrogen oxides
PM	Particulate matter
PCCI	Pre-mixed-charged compression ignition
RCCI	Reactivity-controlled compression ignition
